# The Efficacy of a 3β-Hydroxysteroid Dehydrogenase Inhibitor for the Termination of Mid-Term Pregnancies in Dogs

**DOI:** 10.3390/ani12182475

**Published:** 2022-09-19

**Authors:** Firdevs Binli, İpek İnan, Fatih Büyükbudak, Aykut Gram, Duygu Kaya, Narin Liman, Selim Aslan, Murat Fındık, Serhan Serhat Ay

**Affiliations:** 1 Department of Obstetrics and Gynecology, Faculty of Veterinary Medicine, Ondokuz Mayıs University, Samsun 55280, Turkey; 2 Department of Histology and Embryology, Faculty of Veterinary Medicine, Erciyes University, Kayseri 38280, Turkey; 3 Department of Obstetrics and Gynecology, Faculty of Veterinary Medicine, Kafkas University, Kars 36000, Turkey; 4 Department of Obstetrics and Gynecology, Faculty of Veterinary Medicine, Near East University, Nicosia 99138, Cyprus

**Keywords:** dog, pregnancy termination, 3β-HSD, trilostane

## Abstract

**Simple Summary:**

The medical termination of unwanted pregnancies in dogs is practiced throughout the world for many reasons, including at the request of animal owners. For these procedures, it is advised to use rapidly effective drugs with minimal side effects. In this study, we investigated trilostane, which decreases progesterone levels, for its efficacy in terminating mid-term pregnancies in dogs, as well as potential side effects. Although trilostane is not a standalone alternative for the termination of pregnancy in dogs, it has been determined that its combined use with another medical agent of known efficacy reduces both the abortion time and potential side effects. Further studies investigating an increased frequency of administration rather than the administration dose could contribute to determining the efficacy of trilostane in dogs.

**Abstract:**

Progesterone (P4) is the only hormone needed to maintain pregnancy in dogs. Therefore, a competitive inhibitor of 3β-hydroxysteroid dehydrogenase (3β-HSD) could be a safe and effective option to terminate pregnancy by inhibiting P4 synthesis. To address this hypothesis, we investigated the efficacy of trilostane (TRL), a competitive inhibitor of 3β-HSD, in terminating pregnancy in dogs. Twenty-one dogs between days 30 and 38 of pregnancy were randomly assigned to one of two treatment groups (trilostane (TRL) and aglepristone (AGL)) and an untreated control (CON) group (n = 7 dogs each). Fetal heart rates (FHRs) (measured at 12 h intervals) and serum P4 concentrations (measured at 6 h intervals) were evaluated. The pregnancy termination rates were 0% and 100% in the TRL and AGL groups, respectively. The decrease in the FHR in the TRL and AGL groups was significantly lower than that observed in the CON group. There was a marked decrease in P4 concentrations in the TRL group 6, 54, and 102 h after the initiation of treatment. The luteal expression of StAR appeared to be weaker in the AGL group than the CON group. In conclusion, although a treatment-induced decrease was observed in plasma P4 concentrations, a seven-day TRL treatment alone was not effective in terminating pregnancies. Further studies are needed on the effects of the prolonged administration of TRL with varying doses and frequencies for the termination of mid-term pregnancy in dogs.

## 1. Introduction

**Figure 1:**
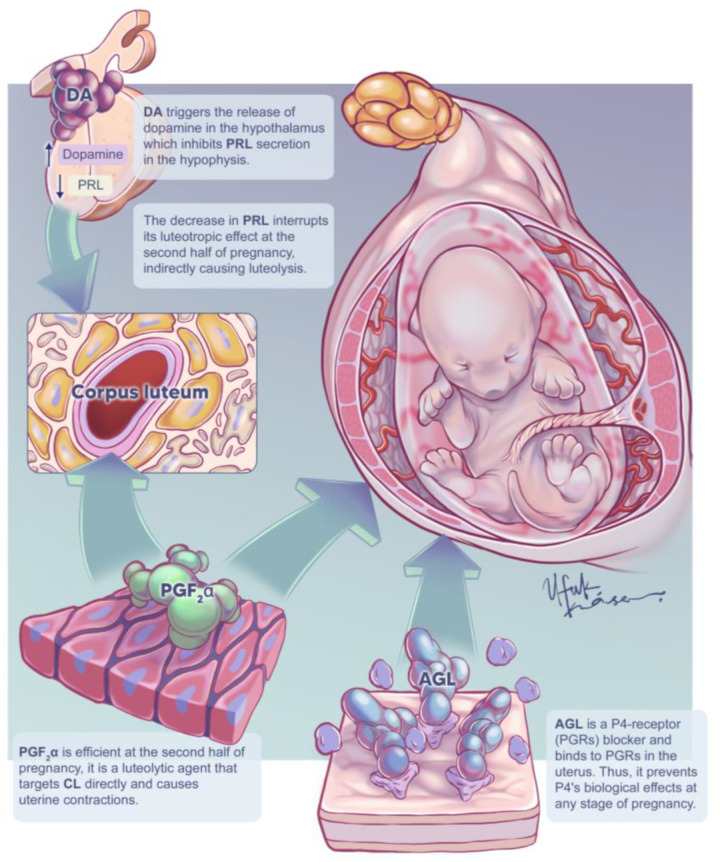
Summary of the abortifacient mechanism of action of PGF_2_α, dopamine agonists (DAs), and aglepristone (AGL).

Progesterone (P4) is the primary hormone responsible for maintaining pregnancy, and corpora lutea (CLs) are the only source of this hormone in dogs [1]. Canine luteal function depends on pituitary support, and luteinizing hormone (LH) and prolactin (PRL) are responsible for maintaining the CL after the second half of pregnancy in dogs [2]. This makes P4 the main target for the medical termination of unwanted pregnancies. Luteolytic agents, such as PGF_2_α, target CL directly [2], whereas dopamine agonists (DAs), such as cabergoline and bromocriptine, target CL indirectly by eliminating PRL support [2]. P4-receptor (PGR) blockers, such as aglepristone, bind to PGRs and prevent P4 activity [3]. The abortifacient effect of PGF_2_α, DA, and PGR blockers are shown in Figure 1.

However, multiple side effects limit the use of these drugs for the termination of pregnancies in dogs [4,5,6]. The main disadvantages of using PGF_2_α for pregnancy termination (PT) in bitches are the side effects and duration of treatment [2,4]. PGF_2_α affects smooth muscles; therefore, bitches that receive PGF_2_α may exhibit emesis, diarrhea, hypersalivation, hyperventilation, abdominal cramps, and transient hypothermia. Side effects depend on the dose administered. Consequently, PGF_2_α is not recommended for use in bitches with cardiopulmonary dysfunction [7]. Furthermore, it does not induce luteolysis, especially during the early luteal phase, and needs to be administered in multiple doses over several days to do so due to the resistance of canine CL to PGF_2_α during the second half of pregnancy [2,4]. The success of PGF_2_α treatment depends on the administration dose, duration of treatment, and the trimester of pregnancy [2]. In addition, PGF_2_α treatment must be continued until PT is completed [4]. Although the termination of pregnancies usually accomplished in between 4 and 7 days, it can take up to 9 days, and live offspring can still be formed after this point [4].

Prolactin secretion is stimulated by serotonin and inhibited by dopamine. The use of DAs causes the release of dopamine, leading to the inhibition of PRL secretion. In this way, they indirectly interrupt the luteotropic effect of PRL [2]. Bromocriptine and cabergoline are two strong dopamine D2-receptor agonists that stimulate the chemoreceptive trigger zone. Bromocriptine D2-receptor activity is not as specific as that of cabergoline and thus has comparatively more severe side effects, such as anorexia, vomiting, and diarrhea [2,8]. In contrast, the side effects of cabergoline are negligible, although both compounds induce insufficient smooth muscle contraction [9]. This feature is the major disadvantage of DAs and causes prolonged fetal expulsion, which is completed within 5 to 7 days [4,5,6]. Notwithstanding, an ideal drug is expected to effectively solve the targeted problems with only minimal side effects [4].

In recent years, aglepristone (AGL), a PGRs blocker, has been widely used for PT in dogs. It can be used for PT from day 0 to 45 (ethical limit) after mating [3]. AGL is a competitive P4 antagonist that binds to PGRs without stimulating the P4-associated molecular sequence [1]. The binding of AGL to PGRs is species- and target-tissue-dependent and 3.12 times greater than that of P4 in dogs [4]. If used for PT, AGL yields higher rates of success when administered within 25 d of ovulation (100%) or 25-45 d after ovulation (95%) [10,11]. However, the use of AGL in early pregnancy is associated with several side effects, such as local pain and itching at the injection site, depression, anorexia, restlessness, and mucoid vaginal discharge [3]. Furthermore, gastrointestinal disorders, tachypnea, and in some cases, endometritis are also encountered in mid-term PT with AGL [1]. Although AGL is a favorable alternative for most of the problems mentioned above, there is still a need for further studies on novel PT methods that produce rapid results without any side effects. In this context, a novel option is to disrupt the synthesis of P4 and prevent its release.

**Figure 2:**
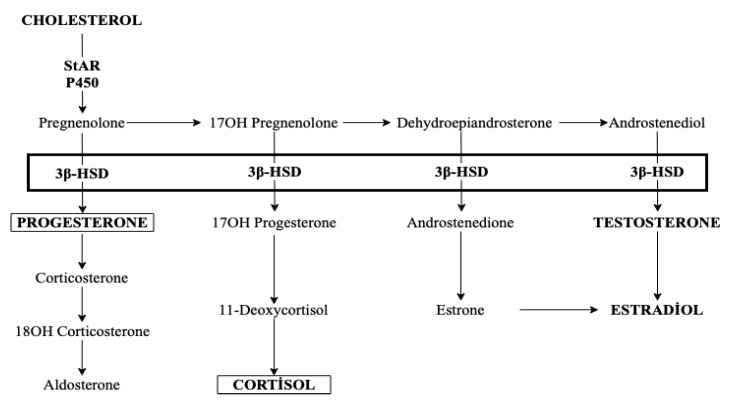
Diagrammatic representation of steroidogenesis.

Steroidogenesis, CL formation, and luteolysis are highly complex processes. Multiple factors and cofactors, including inflammation mediators, enzymes, and luteotropic hormone (PRL), cooperate with the steroidogenic acute regulatory (StAR) protein and 3β-HSD enzymes during P4 production in the canine CL [12]. Cholesterol is the main precursor of all steroid hormones. The first step of steroidogenesis is the transport of cholesterol from the outer to the inner mitochondrial membrane by means of the StAR protein. Transported cholesterol is converted to pregnenolone by the P450 side-chain cleavage located on the inner mitochondrial membrane. The subsequent conversion of pregnenolone to P4 is catalyzed by 3β-HSD in the smooth endoplasmic reticulum [12,13]. Consequently, P4 production is regulated by StAR and 3β-HSD expression levels, and any disruption in the expression of 3β-HSD affects P4 synthesis [12,14] (Figure 2). Thus, inhibiting steroid synthesis or inducing a downregulation in the expression of StAR and 3β-HSD could be an option for PT in dogs.

Trilostane (TRL) is a competitive inhibitor of 3β-HSD, and its use is reported to be safe and effective for the treatment of pituitary/adrenal-dependent hyperadrenocorticism (HAC) and alopecia X in dogs [15]. To date, only three gynecological studies have been performed on dogs using TRL. In these studies, De Bosschere et al. [16] reported that TRL treatment decreased the endometrial wall thickness in dogs with cystic endometrial hyperplasia; De Gier et al. [17] determined that TRL treatment for seven days decreased P4 concentrations in diestric dogs; and Jurzack et al. [18] reported that TRL treatment concurrent with deslorelin administration did not alter ovarian functions. In addition to these studies, the effects of TRL treatment on PT and P4 concentrations have also been investigated in ewes [19,20] and mares [21]. Hence, further studies are needed to establish and/or re-evaluate the effects of the inhibition of the 3β-HSD enzyme. Based on these data, we hypothesized that TRL could be a medical option for PT in dogs by reducing the expression of StAR and 3β-HSD and, consequently, P4 concentrations. Thus, the objective of this study was to evaluate the effects of TRL on mid-term PT in dogs based on clinical findings and hormone and receptor levels.

## 2. Materials and Methods

### 2.1. Animals

The study enrolled 21 dogs of different breeds with unwanted pregnancies. Of these dogs, eleven were mixed-breed, three were Dobermans, three were Setters, one was a Chow, one was a Kurzhaar, one was a Drahthaar, and one was a Dogo Argentino. The mean age of the dogs was 23.25 ± 10.7 months (range 12–28 months), and the mean body weight (BW) was 17.24 ± 5.68 kg (range: 8.65–27.85 kg).

### 2.2. Experimental Design

Bitches with unwanted pregnancy underwent reproductive ultrasonographic and general health examinations.

Transabdominal ultrasonography was performed for both pregnancy diagnosis and the determination of gestational age using a 5–7.5 MHz convex transducer (MyLab™Five VET, Esaote). Gestational age was determined using reference formulae including fetal structures, the crown–rump length, body diameter, biparietal diameter, and internal diameter of the gestational sac [22].

The animals included in the study presented with good general health status and normal pregnancies between 30 and 38 d, as confirmed by ultrasonography and routine clinical assessments, including a CBC and serum biochemistry panel. The bitches were randomly allocated to one of the following groups: trilostane (TRL; n = 7), aglepristone (AGL; n = 7), or control (CON; n = 7).

Informed consent forms were obtained from the owners of all animals included in the study. Each dog was housed in a separate hospital room, fed a standard commercial dog food twice daily, and provided with water ad libitum throughout the study. After the final clinical assessments, the dogs were delivered to their owners.

The experimental design of the study is presented in Table 1. All animals underwent ultrasonographic and clinical examinations throughout the study, starting from the first treatment/control. Ultrasonographic examinations, including Doppler ultrasonography, were performed at 12 h intervals and enabled the monitoring of the fetal structures, viability, and heartbeat. Fetal heart rates (FHRs) were monitored by Doppler ultrasonography to determine possible fetal distress caused by the administered drugs. The average FHR was calculated by counting the heart rates of four fetuses per pregnancy. An FHR of less than 150 bpm/min was considered an indicator of fetal distress [23].

Vaginoscopy and routine physical examinations, including the measurement of the heart rate (Hr), respiration rate (Rr), capillary refilling time (CRT), body temperature, and body weight, as well as control of the lymph nodes and assessment of hydration status were performed at 12 h intervals, whereas complete blood counts (CBCs) and serum biochemistry measurements were performed on a daily basis. Vaginoscopy was performed to examine the vaginal discharge and cervical opening using a vaginoscope of an appropriate size for the examined bitch.

Blood samples were collected from the cephalic vein of all dogs to determine the effects of the tested drugs on blood parameters, kidney and liver functions, and P4 concentration. CBC and serum biochemistry measurements were performed at 24 h intervals. To determine P4 concentrations, blood samples were collected at 6 h intervals and stored at −20 °C after the extraction of sera until the end of the study.

The animals were observed for 10 to 15 min before each blood sampling to detect any clinical changes, such as food and water intake, and were assessed for their general health condition, including abdominal distention, colic, vomiting, diarrhea, and hydration status.

In the present study, the duration of treatment was limited to seven days due to ethical concerns. Thus, we ensured that none of the dogs exceeded the 45 d gestation period and progressed into late pregnancy. Dogs in which PT started within seven days were identified as PT-positive, and the others were considered to be PT-negative. The observation of a green–brown vaginal discharge during inspection or vaginoscopic examinations was considered a sign of PT having started. The absence of fetuses or fetal structures at ultrasonographic examination was accepted as a sign of PT having ended [24]. PT-positive animals were ovariohysterectomized (OHE) immediately after the end of PT, whereas PT-negative and control animals underwent surgery on the morning of day 8 after the first treatment/control.

### 2.3. Medical Induction of Pregnancy Termination

The administration doses and routes of TRL and AGL used to induce PT were selected on the basis of the manufacturers’ recommendations and previously published reports [17,25]. In the TRL group, trilostane (Vetoryl^®^, Dechra Veterinary Products, Basel, Switzerland) was administered orally for seven consecutive days at a dose of 2.2–6.7 mg/kg BW immediately after the morning feeding. In the AGL group, PT was induced by administering aglepristone (Alizin^®^, Virbac, Carros, France) at a dose of 10 mg/kg BW 2×/24 h apart. Untreated animals served as controls (CON).

### 2.4. Ovariohysterectomy and Tissue Sampling

Ovariohysterectomy was performed with a midline incision under general anesthesia induced by propofol (4 mg/kg) and maintained with isoflurane (2%) [26].

Immediately after OHE, the ovaries and uterine horns were washed with PBS, and the corpora lutea were sliced and fixed in a 10% neutral buffered formalin solution, then sent to the laboratory for histopathology [27].

### 2.5. CBC, Serum Biochemistry, and Progesterone Analyses

CBCs (Mindray, BC-5000Vet Auto Hematology Analyzer, Nanshan, China) covered 15 parameters (wbc, rbc, mono, eos, bas, hgb, hct, mcv, mch, plt, neu, lym, mpv, pdw, and pct), and serum biochemistry analyses (Mindray, BS-120, Chemistry Analyzer, Nanshan, China) covered six parameters related to kidney and liver functions (ALP, ALT, AST, GGT, BUN, and creatinine) [28].

P4 concentrations were analyzed using an electrochemiluminescence immunoassay (ECLIA) (Roche Modular E170 Immunoassay Analyzer^®^), as described by the manufacturer, at an internationally accredited laboratory (TÜRKAK, TS EN ISO/IEC 17025:2005). The extraction efficiency was >95%. The mean intra- and interassay coefficients of variance for P4 were 3.2% and 1.7%, respectively. The analytic sensitivity was 0.03 ng/mL [8,24].

### 2.6. Immunohistochemistry

Tissues were collected and preserved as described by Kowalewski et al. [27]. After routine deparaffinization, sections (4-µm-thick) were incubated in citrate buffer (pH 6.0) for 3 × 5 min in a 600 W microwave oven for antigen retrieval. After being cooled to room temperature, the sections were incubated in 0.3% hydrogen peroxide in methanol for 20 min to eliminate endogenous peroxidase activity. After being washed with phosphate-buffered saline (PBS, pH 7.4), the sections were incubated with Ultra V Block (Thermo Fisher Scientific Lab Vision Corporation, Fremont, CA, USA; TA-125UB) for 5 min at room temperature to block non-specific antibody binding. Next, primary antibodies were applied to the sections and incubated at 4 °C overnight. The primary antibodies used were rabbit polyclonal antibody anti-StAR (Cell Signaling Technology, Danvers, MA, USA) and rabbit polyclonal antibody anti-3β-HSD (Dr. J.I. Mason’s gift from Edinburg University). As a negative control, PBS was applied to the sections instead of the primary antibody. Additionally, as an isotype control, non-immune IgGs of the same species, from which the primary antibodies were obtained, were applied at the same protein concentration.

After incubation with primary antibodies, the sections were washed in PBS for 4 × 5 min, incubated with biotinylated secondary antibody (Ultravision Detection System/HRP, Thermo Fisher Scientific Lab Vision TR-125-HL) for 20 min, and washed in PBS for 4 × 5 min. The sections were treated with streptavidin-peroxidase (Ultravision Detection System/HRP, Thermo Fisher Scientific Lab Vision TR-125-HL) for 20 min at room temperature. Peroxide activity was demonstrated using the 3,3-diaminobenzidine chromogen (DAB; Thermo Fisher Scientific Lab Vision Corporation, Freemont, CA, USA) for 5 min. Next, the sections were counterstained with Gill’s hematoxylin for 3 min and washed under tap water until they turned blue. Following staining, the sections were dehydrated through a series of progressively increasing concentrations of alcohol and cleared in xylol. Finally, all sections were mounted in Entellan^®^.

### 2.7. Statistical Analysis

Results are presented as means ± standard errors (SEs), with ranges in parentheses. The Kolmogorov–Smirnov test was used to evaluate the normality of data distribution, and the chi-square test was applied for non-normally distributed features. One-way analysis of variance (ANOVA) was used to compare the fetal heart rates and blood P4 concentrations of the treatment groups (TRL and AGL) and the CON group. Duncan’s test was used to determine the differences between the groups. In addition, ANOVA was used to compare the daily CBC and serum biochemistry values of the control and treatment groups from day 1 to day 8. In the group comparisons, the Bonferroni test was performed to identify the group that made a difference between the averages. The research data were analyzed using the SPSS software Ver. 21.0 (IBM Corp., Armonk, NY, USA).

## 3. Results

### 3.1. Study Groups and Pregnancy Termination Rate

Study group data, including the mean gestational ages, ages, and body weights of the animals, are presented in Table 2. No statistically significant difference was observed between the groups at the beginning of the study.

Pregnancy terminations occurred neither in the animals that received TRL (0/7) nor in the animals that served as controls (0/7). On the other hand, PT occurred in seven out of seven bitches that received AGL (100%). PT started 60 h after the first AGL treatment. The interval of induction to start of PT was 77.71 ± 19.30 h; the interval of induction to end of PT was 114.42 ± 26.17 h; and the duration of PT was 36.71 ± 23.82 h. In four of seven bitches (57.14%), PT was completed on the 4th day. In the remaining three of seven bitches, PT was completed on the 5th (1/7), 6th (1/7), and 7th (1/7) days, respectively.

### 3.2. Clinical Findings and Side Effects

No life-threatening situations occurred in the animals throughout the study. Furthermore, no significant changes were determined in Hr, Rr, CRT, body temperature, or body weight during routine clinical examinations. After the study procedures were completed, all dogs were delivered to their owners in a healthy condition.

Fluctuations observed in the CBC parameters were insignificant, and the measured values were always within the reference intervals in all groups throughout the study. A similar result was obtained with respect to serum biochemistry profiles, excluding ALP activity. ALP activity was lower in the animals treated with TRL (between 43.4 and 49.4 U/L) and AGL (between 22.9 and 45.3 U/L) compared to the control animals (between 75.8 and 93.9 U/L) on days 5, 6, and 7 (*p* < 0.05) but still fell within the reference interval.

No cervical opening was observed in the bitches that received TRL (0/7) or the control animals (0/7) during vaginoscopic examinations. However, when compared to the controls, all animals that received TRL displayed softening of the cervical os upon post-OHE manual macroscopic examination of the removed ovaries and uterus. Although the cervix was closed in the animals treated with TRL, five of seven (71.43%) TRL-treated bitches showed vaginal discharge. Vaginal discharge appeared within 12 to 48 h after the first TRL treatment and disappeared within 12 to 24 h. In contrast to the TRL group, cervical opening with vaginal discharge was observed in all seven animals treated with AGL. In this group, vaginal discharges were of various colors, i.e., yellowish-green (4/7), greenish-brown (2/7), and brown (1/7). Vaginal discharge began to appear within 24 h after the first AGL treatment and continued until the day of OHE.

Trilostane and AGL treatments were well-tolerated. Mild and transient side effects were observed in only two animals treated with TRL (2/7). No side effects were detected in the other groups. One animal vomited on the day of the first treatment (day 0) and showed abdominal tension and pain, and another animal vomited on the last day of treatment. In both animals, the side effects occurred five hours after drug administration. According to the Glasgow pain scale (<4), the use of an analgesic agent was not required in these dogs.

### 3.3. Fetal Distress

There was no statistically significant difference between the groups with respect to the mean FHR immediately before the first treatment/control hour. However, a significant decrease starting 36 h after the first treatment/control was determined in the TRL and AGL groups compared to the CON group (Figure 1).

**Figure 3:**
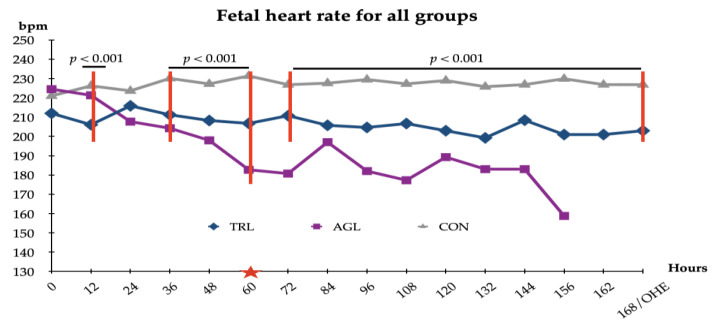
Fetal heart rate (FHR) for all study groups. TRL: trilostane; AGL: aglepristone; CON: control. The red star indicates the start of PT in the AGL group.

During the period from the first count to the last count, the FHR of the CON group was almost constant (from 220.96 to 231.32 bpm/min; *p* > 0.05), whereas significant changes were observed in the TRL (from 211.92 to 200.92 bpm/min, *p* < 0.01) and AGL (from 224.46 to 158.75 bpm/min, *p* < 0.001) groups. On the other hand, despite the significant decrease observed in the FHRs of the treatment groups, their mean FHR values remained above the fetal distress level (Figure 3).

### 3.4. Plasma Progesterone Concentrations

**Figure 4:**
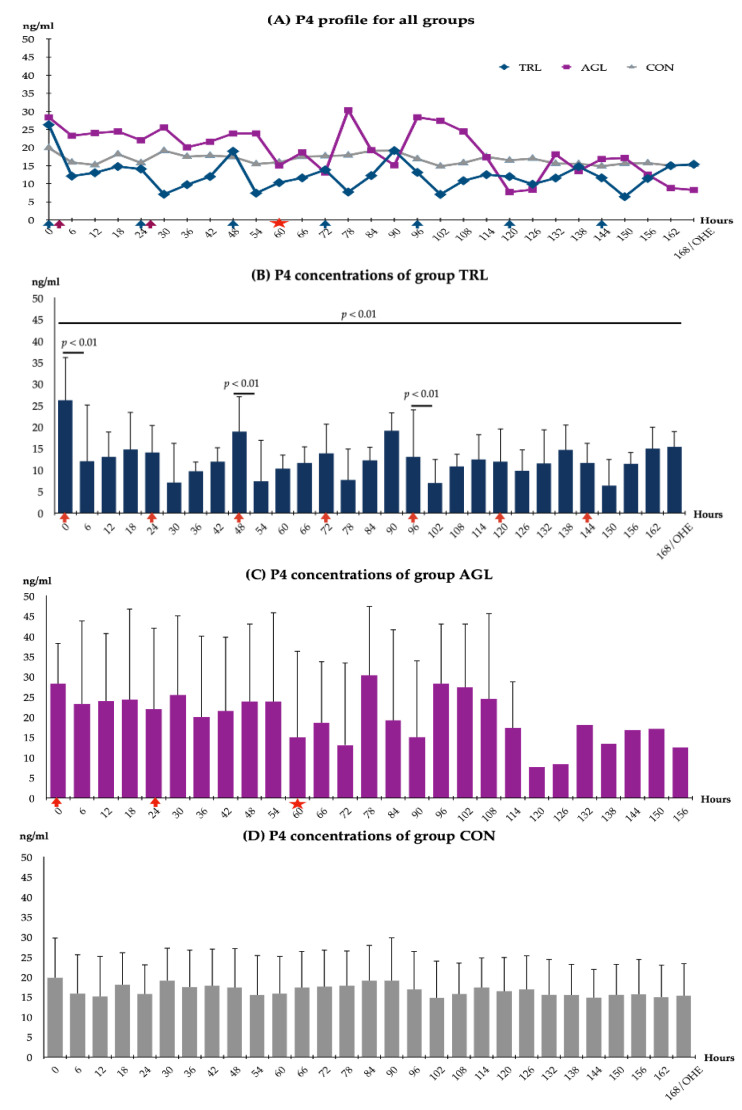
Serum P4 profiles for all groups. TRL: trilostane; AGL: aglepristone; CON: control. (**A**) and P4 concentrations for each of the groups (**B**–**D**). TRL and AGL treatment time indicated by dark blue and purple arrows (**A**) and red arrows (**B**,**C**). The red star marks the start of PT in the AGL group in subfigures (**A**,**C**). Statistical analysis was not performed in the AGL group, as the number of animals decreased after 60 h due to PT (**C**).

The P4 profiles of the study groups are presented in Figure 4A. There was no statistically significant difference between the groups in terms of P4 concentrations. However, fluctuations were observed in both the TRL group and the AGL group. In the CON group, P4 concentrations remained constant.

**Figure 5:**
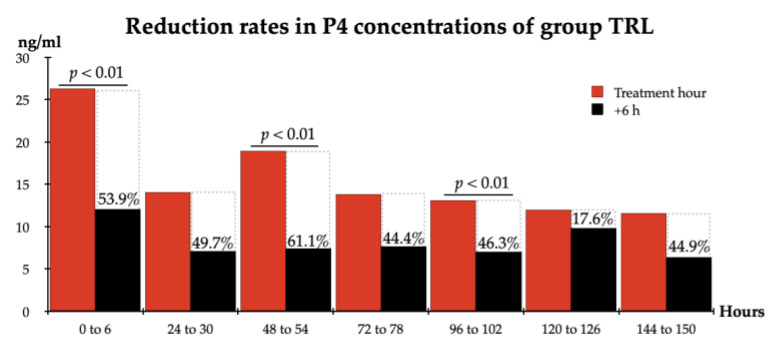
Reduction rates in P4 concentrations 6 h after each TRL treatment. TRL: trilostane; AGL: aglepristone; CON: control.

In the TRL group, an almost 61.1% reduction in P4 concentrations was achieved from the start of the treatment (0-h) to the end of the study (168 h/OHE) (from 26.27 ng/mL to 10.23 ng/mL; *p* < 0.05) (Figure 4B). A significant decrease was observed in P4 concentrations within six hours after each of the TRL treatments, such that even within the first six hours, a 53.97% reduction was achieved (from 26.27 ng/mL to 12.09 ng/mL; *p* < 0.01). Furthermore, reduction rates of 49.7% (h30), 61.1% (h54), 44.4% (h78), 46.3% (h102), and 44.9% (h150) were reported in P4 concentrations within six hours after each TRL treatment, except for 120 h (Figure 5). Moreover, at these time points, P4 concentrations were at the lowest levels measured in the study. Compared to the initial levels measured at the beginning of the study (26.27 ng/mL), the rates of decrease in the P4 concentrations at these time points were as follows: 73.1% at 30 h (7.07 ng/mL), 71.9% at 54 h (7.38 ng/mL), 70.7% at 78 h (7.69 ng/mL), 73.2% at 102 h (7.03 ng/mL), and 75.7% at 150 h (6.39 ng/mL) (Figure 4B).

In The AGL group, due to the decrease in the number of animals (n) as a result of the start of PT, a statistical comparison of the P4 concentrations was only performed up to 60 h; furthermore, there was no significant difference between the P4 concentrations measured at the various time points (*p* > 0.05) (Figure 4C). Minor changes in the P4 concentrations of the CON group were not statistically significant (Figure 4D).

### 3.5. Expression of StAR and 3β-HSD in Canine Corpora Lutea

**Figure 6:**
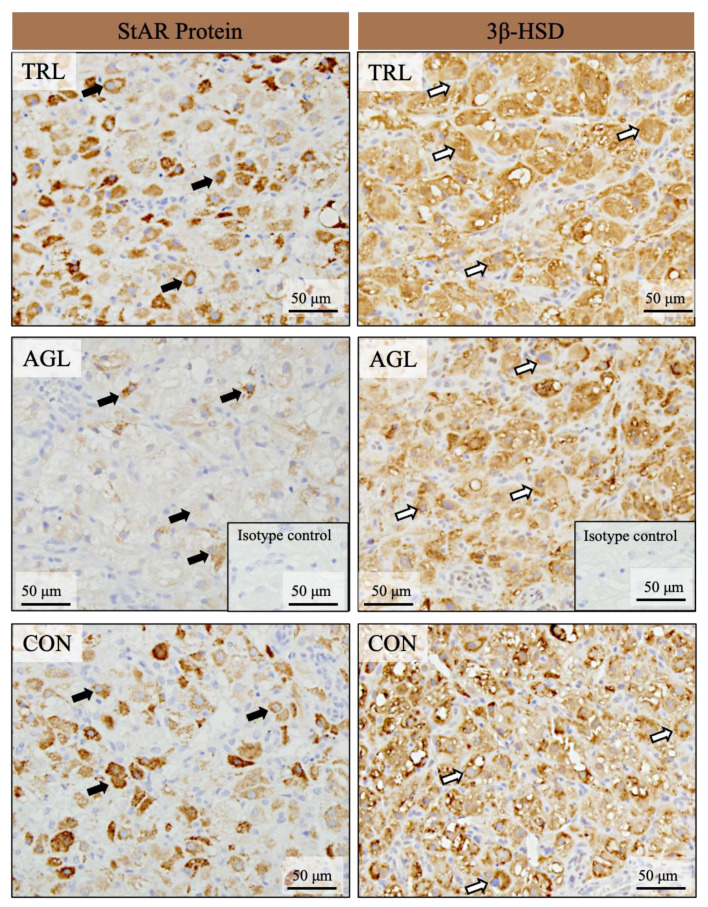
Localization of StAR protein (black arrows in the left column) and 3β-HSD (white arrows in the right column) in the luteal cells, as demonstrated by immunohistochemical staining. TRL, trilostane; AGL, aglepristone; CON, control.

StAR and 3β-HSD were expressed in the CL of all of the dogs included in this study. Their expression was detected only in the luteal cells but not in the other cell types of the CL, such as endothelial cells or connective tissue cells. The intensity of StAR and 3β-HSD staining in the luteal cells of the AGL-treated dogs appeared weaker than that observed in the control dogs. These findings in the AGL group were observed the day after the end of PT. In the TRL group, the intensity of StAR and 3β-HSD staining seemed similar to that of the CON group (Figure 6). The PT completion times are presented in Table 2.

## 4. Discussion

Our results clearly show that TRL treatment does not induce PT in mid-term pregnant dogs, although it causes a significant decrease in P4 concentrations, at least when administered alone and at the dose recommended by the manufacturer.

Whereas TRL is licensed for the treatment of Cushing’s disease in dogs [29], the aim of this study was to suppress the synthesis of P4, not cortisol. However, considering the steroid hormone synthesis pathway (see Figure 2), it is possible that TRL suppresses cortisol production in an attempt to suppress P4 production. In this study, the adrenocorticotropic hormone (ACTH) stimulation test, suggested as a gold standard by some authors for pretreatment assessment [30], was not performed before or during treatment. Therefore, there is a need to explain why the ACTH stimulation test was not performed in the present study. First, there is no consensus with respect to the requirement to perform the ACTH stimulation test in HAC treatment [31]. It has been reported that there is a need for new methods to replace the ACTH stimulation test [32], given its disadvantages, including frequent false-positive or false-negative results, high cost, and the results being influenced by the presence of a chronic illness or stress [31]. Secondly, it is recommended to perform the ACTH stimulation test 10 to 14 d after the first TRL treatment [29]. Based on these data, the ACTH stimulation test was not performed in this study in order to avoid the possibility of affecting the results, such as the induction of PT.

On the other hand, as previously described in the literature, in this study, animals were evaluated in terms of clinical findings of hypercortisolism, including polyuria/polydipsia, shaking, vomiting with CBC (nonregenerative anemia and absence of stress leucogram), and serum biochemistry, together with routine clinical parameters [33].

### 4.1. Routine Clinical Findings

The expectation of an abortifacient drug is that it has no side effects and does not adversely affect future fertility [4]. Trilostane has been reported to be relatively safe and effective for the treatment of HAC in dogs [29] and for PT in women [34], and this is especially true for low doses of TRL in dogs and humans [29,34]. On the other hand, side effects of varying incidence and severity have been observed in both animals and humans following TRL administration [29,33,34,35,36,37,38]. In dogs treated for HAC, the most common side effects of TRL have been reported as self-limiting lethargy, vomiting, and diarrhea [36]. Furthermore, treatment-induced mortality is possible due to the excessive suppression of the adrenal glands, although such cases are relatively rare [37]. Lemetayer and Blois [29] reported side effects in 13% of dogs receiving TRL at 24 h intervals and in 19% of dogs receiving TRL at 12 h intervals for HAC treatment. However, it is also known that the incidence of side effects can increase by up to 40% [15,33] and even 63% [39] in dogs treated for HAC. In women treated with TRL for PT, vomiting has been reported as the most common side effect after symptoms such as burning, itching, and a flushing feeling in the face [34,35,38].

The side effects generally resolve spontaneously within 24 to 48 h of drug withdrawal [15,29,33,40,41]. The reports mentioned above may explain the low and mild incidence of side effects observed in the present study.

Although the side effects of TRL tend to increase with increased dose, frequency, and duration of the treatment [42], they may also develop in opposite situations, such as treatment at a low dose (1 mg/kg/12 h) and for a short time (seven days) [29]. Therefore, it has been reported that other factors, apart from an increased dose, frequency, and duration of treatment, may also lead to the appearance of side effects [29]. In view of these data, we speculated that the side effects observed in this study appeared as a result of the interaction of the drug with the biochemical and physiological changes caused by pregnancy.

Aglepristone is a safe abortifacient agent in dogs and cats [3,43]. Its side effects were briefly mentioned in the introduction. Bitches treated with AGL for mid-term PT (25 to 45 d of ovulation) have been reported to show depression and anorexia at rates of 20.3% (14/69) [44] and 36.4% (8/22), respectively [25], and gastrointestinal disturbance at a rate of 13% (9/69) [44]. These side effects also were reported to be mild, transient, and negligible [3].

Pregnancies are terminated via both resorption and abortion after the second half of pregnancy [3]. Therefore, in this study, vaginal discharge resulting from the separation of the uteroplacental structures during PT was considered a normal clinical sign and not a side effect. Furthermore, vaginal discharge indicates the start of PT [24]. This presentation can vary in character, color, and incidence, depending on the stage of pregnancy. For example, such discharge can be mucoid [43] or serosanguinous [2] in character and yellowish-green or brown in color. It has been reported to have been observed at incidences of 87.5% [24], 31.88% [45], and 77.27% [25]. Fetal extraction is more expected after vaginal discharge than resorption [43,44]. Therefore, cervical ripening is also important for PT during the second half of pregnancy.

There have been no reports of vaginal discharge during or after TRL treatment in dogs with HAC or alopecia X. However, published studies have not been conducted on pregnant dogs. Vaginal discharge of varying character, color, and incidence has been reported in previous studies on the use of epostane or TRL in association with the induction of parturition or PT in humans, Rhesus monkeys, and sheep [19,41,45]. However, vaginal discharge has not been reported in diestric [17] or anestric dogs [18]. On the other hand, we observed serous vaginal discharge for 12 to 24 h in five of seven bitches in the TRL group in the present study.

Furthermore, upon post-OHE examination, a softening was determined in the cervical os of all animals that received TRL treatment. It is well known that there is a close correlation between decreased P4 concentrations and cervical ripening before parturition or abortion. Haluska et al. [45] reported that cervical softening occurred within 24 h after a 50% decrease in P4 concentrations induced by epostane treatment in Rhesus monkeys. The origin of vaginal discharge could be the uterus, the cervix, or the vagina itself [46]. Based on the abovementioned literature, we speculated that the source of the serous vaginal discharge observed in the animals treated with TRL in the present study was the cervix. This speculation seems feasible, considering the decrease observed in P4 concentrations after TRL treatment. In bitches, cervical opening is induced by a maximum estrogen:progesterone ratio [47]. However, parturition plasma estrogen concentrations do not increase in bitches but remain stable or decrease [48]. Therefore, decreasing P4 concentrations can result in a high estrogen:progesterone ratio.

The most common clinical sign reported in bitches treated with AGL for mid-term PT is brown hemorrhagic vaginal discharge, including fetal parts, which has been observed at rates of 31.8% [44] and 77.3% [25]. In the present study, vaginal discharge was green-colored rather than brown-colored. However, considering the structure of the canine placenta and the uteroverdin pigment found in bitches [49], vaginal discharge of both colors is normal in bitches with PT.

Some physiological changes may occur in the CBC and serum biochemistry panel during pregnancy, especially in the late stages [50,51]. However, the CBC and serum biochemistry results were within the reference intervals in the present study. This was attributed to the animals being in the early rather than late stage of pregnancy, during which such changes occur [51]. Our results show that TRL and AGL treatment did not cause any significant difference in the CBC and serum biochemistry panel, except for ALP activity. Generally, previous studies on canine PT have not evaluated these parameters.

In the present study, ALP activity was significantly lower in the treatment groups as of the 5th day, compared to the control animals. This significance can be neglected for animals treated with AGL due to the reduced number of animals resulting from PT. On the other hand, decreased ALP activity was an acceptable result for the TRL group, considering that the drug is metabolized in the liver [29]. However, in this study, the duration of treatment was short. Fetal hepatic hematogenesis could be an essential factor in explaining the differences in ALP activity because, according to Kimura and Kotani [51], it may cause an elevation in maternal ALP activity. Therefore, we suggest that TRL may disrupt fetal hepatic hematogenesis and should be further investigated in future studies.

### 4.2. Fetal Heart Rate

It is well known that low embryonic or fetal heart rate indicates fetal distress and is a risk factor for abortion in dogs [52,53]. In this context, we had two goals in monitoring FHR in this study. The first was to determine whether the treatments caused fetal distress, and the second was to estimate the time of the start of PT using these data in cases in which the treatments caused fetal distress.

In dogs, during normal pregnancy, the FHR should be 220 bpm, and FHRs under this level indicate fetal distress, such that 180–220 bpm implies moderate distress, and 150–180 bpm implies severe distress [23,54,55]. Care should be taken to increase the chance of fetal survival. An intervention is required for fetuses with an FHR < 160 bpm, such that fetuses with an FHR < 130 bpm must be delivered within 1 to 2 h, and fetuses with an FHR < 100 bpm must be delivered immediately [55,56].

In the present study, TRL and AGL treatment significantly decreased the FHR. However, despite this decrease, the FHR still remained above the threshold level of fetal distress in the TRL group. Therefore, it would erroneous to conclude that TRL treatment caused fetal distress. However, given a significant decrease having been observed after almost every treatment, TRL treatment may have caused a temporary decrease in the FHR. Based on these data, we can speculate that TRL treatment could induce fetal distress if applied at an increased frequency.

Our results show that the estimated start time of PT could not be determined by FHR monitoring performed at a minimum interval of 12 h. It is clear that further studies are needed to investigate fetal distress levels that account for an estimated start time of abortion. In future studies should consider that temporary fetal bradycardia or tachycardia may develop during pregnancy; therefore, a FHR lower than twice the maternal heart rate should be considered a serious finding of fetal distress, as reported by England [52]. In addition, the FHR should be evaluated together with the fetal unit, as in human medicine [57], and FHR monitoring should be performed more frequently, as reported by Breukelman et al. [53].

### 4.3. Pregnancy Terminations and Progesterone Concentrations

The elimination of the effect of P4 is essential to the induction of parturition/PT. Our results demonstrate that although TRL treatment caused a significant decrease in the peripheral P4 concentrations of mid-term pregnant dogs, it was not able to trigger PT when used alone, at least at the dose recommended by the manufacturer. This supports the notion that P4 synthesis could be disturbed by TRL treatment. However, several publications have reported that low P4 concentrations triggered by epostane and TRL treatment induce parturition/PT [41,58,59]. In women, high-dose epostane was found to terminate more than 83% of early pregnancies [41] within 14 days, whereas in early-pregnant dogs, low-dose epostane was reported to terminate all pregnancies [58]. Similarly, TRL ended 25 d of pregnancy in Rhesus monkeys [59]. Furthermore, with TRL treatment, 66% of pregnancies in sheep were induced for parturition [59], and 100% of 90-day pregnancies were terminated in sheep [20]. The abovementioned literature reports also indicated that the effect of this enzyme inhibitor on P4 concentrations was dose-dependent. On the other hand, two studies—one in mares [60] and one in dogs [17]—support our results, showing that a significant decrease in P4 concentrations triggered by TRL did not cause PT. In the present study, P4 concentrations did not fall below 2 ng/mL, which is essential to the onset of labor in dogs. P4 concentrations only decreased to a level around 7 ng/mL (22.26 nmol/L) following TRL treatment, which is lower than the value reported in a study by De Gier et al. (around 50 nmol/L) [17].

We speculated that a rapid and high level of decrease in the P4 concentration could induce PT before activating the adaptive/compensative mechanism. Both epostane and TRL are capable of fulfilling this purpose. It has been demonstrated that epostane treatment may result in more than a 90% decrease in P4 concentrations within 30 min in sheep and women [40]. Similarly, TRL treatment is also capable of causing a significant decline in P4 concentrations. It is reported to have caused a 65–79% decline in the P4 concentrations of women within 48 h [34,35], a 70% decline in the P4 concentrations of sheep [61], and a 56–68% decline in the P4 concentrations of non-pregnant dogs within 3 d [17]. In TRL-treated dogs, a reduction of approximately 53.97% and 73.08% was detected in P4 concentrations at the 6th and 30th hours after treatment, respectively, which is in agreement with previous reports. In the present study, the decrease in P4 concentrations at 6 h after each treatment was followed by a progressive increase thereafter. This result suggests that the effect of TRL is temporary, as reported in previous publications [29,33]. Thus, the frequency of treatment [29] and/or the administration dose [37,62,63] should be increased to obtain the abortifacient effect of TRL. Moreover, it was reported by De Gier et al. [17] that low doses of epostane are effective in inducing PT [58] and are more effective than the normal administration dose of TRL in reducing P4 concentrations in bitches.

Based on these data and literature reports, there are two main reasons why we recommend increasing the administration frequency or dose of the drug to achieve the desired PT effect. The first is not achieving effective doses in the tissues, and the second is the tissues not responding to the drug.

The first reason for our recommendation is related to the pharmacokinetics of the drug. TRL is an orally active enzyme inhibitor and is poorly soluble in water. Therefore, it should be administered with food to enhance absorption [33]. However, this feature of the drug causes differences in the time to reach the peak level of TRL in the blood and the duration of its action. For example, the peak-level time is 0.5 to 1 h, 2 to 4 h, and 1.5 h in rats, humans, and dogs, respectively. Because TRL and its metabolites do not accumulate in tissues, the duration of action of this drug is less than 20 h. Therefore, its level in the blood returns to the baseline after 12 h [29,33]. Another explanation offered by Jurczak et al. [18] is that TRL may have limited access to steroidogenic cells in the canine ovaries.

The second reason is related to the 3β-HSD enzyme varying among species or tissues. Although the 3β-HSD enzyme of dogs displays 79% to 86% similarity to the enzyme of other species, several isoforms have been identified in different species and tissues [64]. For example, there are six isoforms in humans, four isoforms in rats, and two isoforms in mice. In rats, both type 1 and type 2 are expressed in the adrenal glands [65]. In humans, type 2 is mainly expressed in the gonads and adrenal glands, and type 1 is expressed in trophoblasts, mammary glands, and skin [66]. It has been demonstrated that only the type 1 isoform, and not type 2, is inhibited by low doses of epostane in humans [67]. In dogs, no isoform was found [64,65]. However, according to Kowalewski et al. [64], the 3β-HSD enzyme of dogs has a substrate-specific character similar to that of type 1 in humans and rats. This report is supported by the results of our study and the previous clinical studies mentioned above, which have demonstrated that TRL significantly decreases P4 concentrations in women [34,35] and animals [17].

However, increasing the treatment dose of the drug could increase the possibility of side effects. For example, plasma cortisol concentrations may fall below the safe level in dogs. Therefore, increasing the treatment dose of the drug is not recommended [63].

Another way to increase the effectiveness of TRL or AGL without increasing side effects is to combine their use with other drugs. Combined treatment improves treatment outcomes through synergistic activity, reduces the toxicity or side effects of the treatment/drug regimen, reduces the doses used for treatment, and reduces the emergence of drug resistance [68]. Many combined treatment regimens, such as TRL + misoprostol [35], TRL + PGF_2_α, TRL + cortisol, TRL + PGF_2_α + cortisol [20], AGL + misoprostol [8,24,69], AGL + PGF_2_α [24], and AGL + cabergoline [8], have been used in human and veterinary medicine to induce parturition/PT.

It has been demonstrated that antigestagen treatment performed before prostaglandin treatment increases sensitivity to prostaglandins [34]. These data are supported by multiple studies performed in humans [34,35] and animals [6,8,24,69]. Although no previous study has investigated the combined use of TRL with any other drug to induce parturition/PT in animals, many studies have been conducted on the combined use of AGL with various drugs [6,8,24,69]. The common conclusion of these studies is that combinations are more effective than the administration of a single drug. Two human medical reports [34,35] showed that TRL treatment performed prior to misoprostol treatment shortened the interval between treatment and PT by 50%.

Unlike other abortifacient agents and normal parturition, in AGL-induced parturition/PT, P4 concentrations do not decrease to the basal level and remain high [6,8,24,69]. As an antiprogestin, AGL abolishes the biological effect of P4 by blocking PGRs [70]. Therefore, high P4 concentrations in bitches during AGL treatment can be considered normal. However, whereas some authors report that a significant increase in P4 concentrations occurs after the first AGL treatment [24,28,71,72], others report an insignificant change in P4 concentrations after AGL treatment [43,69,73]. In this study, the change in the P4 concentrations of the bitches treated with AGL was insignificant, and no increase occurred after the first AGL treatment.

Parturition is a complex physiological process involving many factors, such as corticosteroids, P4, PRL, relaxin, and PGF_2_α. On the other hand, the endocrinological processes of parturition are not fully understood in any species [48]. However, it is well known that parturition and PT begin with prepartum luteolysis, which is characterized by a decrease in peripheral P4 concentrations and the downregulation of StAR and 3β-HSD expressions in luteal cells in response to an increase in PGF_2_α levels [13,74]. On the other hand, the decrease in P4 concentrations that triggers the mentioned regulatory pathway does not apply to AGL-induced parturition/PT [24,28,71,72] because AGL causes incomplete luteolysis [73]. Kowalewski et al. [75] showed that blocking PGRs by AGL leads to the activation of the prostaglandin system in the luteal and placental tissues, with a similar occurrence during normal prepartal luteolysis. Although there is a need for further molecular studies on this subject, the aforementioned studies explain the P4 and PT data we obtained in the present study.

### 4.4. Expression of StAR and 3β-HSD in Canine Corpora Lutea

The role of the StAR protein and the 3β-HSD enzyme in progesterone synthesis was summarized in the Introduction section (see Figure 2). Both the StAR protein and the 3β-HSD enzyme are expressed in the cytoplasm of canine luteal cells, and the P4 profile is consistent with the expression levels of these factors in the CL during diestrus and pregnancy [12].

Many paracrine and/or autocrine mechanisms control luteal functions [76]. For example, in the second half of the CL life span, PRL, the luteinizing hormone (LH) [74], prostaglandin E2 (PGE2), and P4 itself [27] come into play as luteotrophic factors to provide the high level of P4 necessary for the continuation of pregnancy.

Trilostane treatment changed the expression of neither StAR nor 3β-HSD in luteal cells but caused a significant decrease in P4 concentrations. To the best of our knowledge, this is the first study to evaluate the effect of TRL treatment on StAR and 3β-HSD expressions in luteal cells by immunohistochemistry in dogs. Therefore, the lack of studies on this subject has made it difficult to discuss the obtained results. However, we still presented comments in the light of available literature data. We focused on two significant hypotheses concerning why the expressions of these factors did not change. The first hypothesis is that whereas TRL treatment causes a reduction in the expression of StAR and 3β-HSD, this reduction is temporary, and the expression levels later return to normal. A previous study in rats supports this possibility, as PGF_2_α treatment caused a 50% reduction in 3β-HSD expression, which returned to normal after 8 h [77]. This suggests that TRL treatment can temporarily alter the number of receptors, even at an hourly level. We consider the second hypothesis to be more plausible. As is well-known, the effect of the 3β-HSD enzyme inhibitor is temporary [29], and the activity of 3β-HSD could be affected by endogenous or circulating P4 concentrations [78], especially in the absence of adaptive/compensative factors, such as substrate activation and the extrinsic and intrinsic luteotrophic factors mentioned above. Therefore, we considered that TRL shows its effect by preventing enzymatic activity at the substrate level [64] but not during the secretion process. This may also explain the varying catalase activity of the drug in tissues [79]. Although dogs do not have different isoforms of the enzyme [64], at least in the luteal tissue, TRL may induce a higher level of catalase activity in the adrenal tissue than in the luteal tissue [80]. In addition, luteotrophic factors similar to those found in dogs, including LH/hCG, LH, and PRL, have also been determined in humans [81] and rats [82]. It has been shown that the activation of these factors is critical to the maintenance of 3β-HSD expression in both species. Furthermore, it was demonstrated by De Gier et al. [17] that TRL treatment has no effect on circulating PRL concentrations in dogs. In this context, intrinsic luteotrophic factors, such as growth factors [83], which play an autocrine role in the regulation of 3β-HSD, are also important.

As briefly mentioned in Section 4.3, AGL causes incomplete luteolysis without decreasing P4 concentrations and initiates the prepartal luteolytic cascade-like process observed during normal parturition [12,13,73,74,75]. Based on these studies, the downregulation of both StAR and 3β-HSD expression can be considered normal in the AGL group.

Although the number of dogs enrolled in this study is relatively small, based on the data obtained in the study, we speculate that TRL is not able to trigger a cumulative effect to cause luteolysis in bitches. Further studies using molecular techniques are needed to identify and fully elucidate the effect of TRL treatment on StAR and 3β-HSD expression.

## 5. Conclusions

Although we could not confirm our hypothesis in this study, we achieved promising results for future studies. The results of the present study indicate that (1) TRL is able to cause a significant decrease in P4 concentrations but is not able to trigger PT, at least at the dose and frequency of treatment recommended by the manufacturer; (2) TRL treatment for the induction of PT causes insignificant side effects that can be neglected by practitioners; (3) TRL treatment caused softening in the cervix, even if it did not cause cervical opening; and (4) the decrease in P4 concentrations is not related to StAR and 3β-HSD expressions in luteal cells. There is also the possibility of inducing PT if the duration of TRL treatment is extended. However, we believe that extending the treatment for a further seven days to induce PT has no practical value. Based on the promising results mentioned above, we suggest that further clinical and molecular studies should be designed to further elucidate the abortifacient effect of TRL in dogs. Based on available literature reports, we have some suggestions. First, when administered at a high dose and/or frequency, the inhibitory effect of TRL on steroid synthesis increases [29,37,62,63]. However, such a treatment regimen is not recommended by some authors, as it may increase the incidence and severity of side effects. Considering the sharp decline in P4 concentrations at the 6th hour after treatment, it seems logical to increase the frequency of treatment rather than the administration dose. Thereby, a more dramatic decline in the P4 concentration can be achieved to induce cervical opening and result in PT.

Furthermore, such a treatment regimen could cause fewer side effects than expected from a high-dose treatment [29]. Secondly, we believe that if TRL treatment is combined with another abortifacient agent, such as PGF_2_α, cabergoline, or AGL, a satisfactory PT rate associated with milder and fewer side effects can be achieved.

## Figures and Tables

**Table 1:** Experimental design of the study.

Days Hours		d1 0	6	12	18	d2 24	30	36	42	d3 48	54	60	66	d4 72	78	84	90	d5 96	102	108	114	d6 120	126	132	138	d7 144	150	156	162	d8 OHE
Treatment	TRL	•				•				•				•				•				•				•				
	AGL	•				•																								
Clinical assessments		•		•		•		•		•		•		•		•		•		•		•		•		•		•		•
CBC & Biochemistry		•				•				•				•				•				•				•				•
P4 assay		•	•	•	•	•	•	•	•	•	•	•	•	•	•	•	•	•	•	•	•	•	•	•	•	•	•	•	•	•

**Table 2:** Mean gestational age, age, body weight, PT rate, and average PT duration of the dogs in the study groups.

Group	GA (d; *X* ± *SE*)	Age (m; *X* ± *SE*)	Body Weight (kg; *X* ± *SE*)	PT	
				Rate (%)	Duration (h; *X* ± *SE*)
**TRL (n = 7)**	36.1 ± 0.9	22.8 ± 14.9	18.5 ± 6.1	0 (0/7)	-
(range)	(32–38)	(12–48)	(15.2–27.8)		
**AGL (n = 7)**	33.9 ± 1.2	20.6 ± 8.4	16.1 ± 5.1	100 (7/7)	36.7 ± 23.8
(range)	(30–38)	(12–36)	(10–24)		
**CON (n = 7)**	35.4 ± 1.1	25.2 ± 10.7	17.1 ± 6.4	0 (0/7)	-
(range)	(32–38)	(12–48)	(9.7–27)		
***p* value**	*p* > 0.05	*p* > 0.05	*p* > 0.05	-	-

TRL: trilostane, AGL: aglepristone, CON: control, GA: gestational age, PT: pregnancy termination.

## Data Availability

Data sharing is not applicable to this article.
